# Intergenerational differences in turnover intention of nurses: a cross-sectional survey in Jiangsu province, China

**DOI:** 10.3389/fpsyt.2025.1550623

**Published:** 2025-05-15

**Authors:** Guangyu Hu, Zhen Wang, Chaoran Zhang, Jingwen Xu, Ziheng Shen, Lixin Peng, Haibo Xu

**Affiliations:** ^1^ School of Management, Xuzhou Medical University, Xuzhou, China; ^2^ Medical Affairs Department, Affiliated Stomatological Hospital of Xuzhou Medical University, Xuzhou, Jiangsu, China; ^3^ Department of Health and Management, Taishan Vocational College of Nursing, Taian, Shandong, China; ^4^ The Second Clinical Medical School, Xuzhou Medical University, Xuzhou, Jiangsu, China; ^5^ Medical Affairs Department, Affiliated Hospital of Xuzhou Medical University, Xuzhou, Jiangsu, China; ^6^ Center for Mental Health Education and Research, Xuzhou Medical University, Xuzhou, Jiangsu, China

**Keywords:** work-family conflict, social support, resilience, intergenerational differences, nurses

## Abstract

**Purpose:**

This study aimed to learn the turnover intention of nurses in the workplace and analyze the influencing factors, commonalities, and differences from the perspective of intergenerational differences.

**Methods:**

Between 4 September and 5 October 2023, a stratified cluster sampling was conducted among 2299 nurses at 16 tertiary hospitals in Jiangsu Province in China, using the questionnaire composited of General sociodemographic information, Work-Family Conflict Scale, Perceived Social Support Scale, Resilience Scale, and Turnover Intention Scale. SPSS v26.0 was performed to analyze data.

**Results:**

A total of 2112 participants were included. The turnover intention of “Generation X” (born between 1965 and 1980) nurses was lower than that of “Generation Y” (born between 1981 and 1996) and “Generation Z” (born between 1997 and 2012). Work-family conflict was a common influencing factor on the turnover intention of three generations of nurses (P < 0.05). Family-work conflict (β = 0.099, P < 0.001), other support (β = -0.169, P < 0.001), resilience (β = -0.103, P < 0.001), night shifts (β = 0.047, P = 0.033), the number of children (β = -0.054, P = 0.041) and occupational diseases (β = -0.108, P < 0.001) were specific influencing factors of turnover intention among “Generation Y” nurses. Resilience (β= -0.172, P = 0.001) and family support (β = -0.188, P = 0.001) were specific factors of turnover intention in “Generation Z” nurses.

**Conclusion:**

This study reveals the intergenerational differences in the turnover intention of nurses and its influencing factors. The turnover intention of “Generation Y” and “Generation Z” nurses is higher than that of “Generation X”, and work-family conflict is the common factor of their turnover intention. It is suggested that hospital managers formulate targeted intervention strategies to reduce turnover intention according to the intergenerational characteristics of nurses.

## 1 Introduction

The healthcare industry is the largest workforce in the world, and nurses make up a large share of that workforce (1). According to the 2024 China Statistical Yearbook, the number of physicians and nurses in China has reached 4,782,086 and 5,637,142, respectively. As China’s aging population becomes more pronounced, placing greater demands on the nursing profession, the demand for nurses is likely to increase gradually (2), and the shortage of nurses is a critical issue worldwide. The Theory of Planned Behavior indicates that an individual’s intention influences the occurrence of behavior (3). The high turnover intention among nurses ultimately leads to actual turnover behavior, thereby exacerbating the shortage of nursing staff. Previous studies have shown that pay levels, job satisfaction (4), peer support, and work environment (5) all have some impact on nurses’ turnover intentions. In addition, principled ethical climates can reduce turnover intention through trust in the organization (6). Although there have been some cross-sectional surveys of nurses’ turnover intention in different provinces (7, 8), this study highlights the intergenerational difference in nurses’ turnover intention in Jiangsu Province, China.

Generational differences reflect the differences in behavior and values between generations due to changing eras and are based on the group rather than the individual. Currently, the main body of Chinese nursing staff consists of three main generations: “Generation X” (born between 1965 and 1980), “Generation Y” (born between 1981 and 1996), and “Generation Z” (born between 1997 and 2012) (9). A study that examines the work participation of nurses across different generations also revealed that compared to “Generation Y”, “Generation X” demonstrates higher levels of dedication to work (10). A survey of nurses in Ontario showed that the younger generational nurses had significantly lower job satisfaction (11). Job satisfaction was positively correlated with intention to stay indicating that the younger generation is more likely to have a high turnover intention (12). “Generation Y” is the first demographic cohort impacted by the one-child policy. As a generation predominantly composed of only children, many of them face the burden of supporting their elderly parents alone, which can potentially lead to work-family conflicts. Therefore, this study suggests that the need to provide care is a factor that influences willingness to change. “Generation Z” is living in an era of the fastest economic development and an abundance of material goods. This generation is highly adaptable to new technologies and is more proficient in using modern medical equipment. In a study with a follow-up survey, it was shown that reducing the effort to ensure robots’ smooth operation helped to reduce time pressure and thus nurses’ turnover intention (13). Furthermore, nurses of different generations have different professional ethics and occupational cognition, which need to be managed in a specific way and provide them with a suitable work environment and family support. Therefore, it is necessary to study the factors influencing the turnover intention of different generations among nurses.

Work-family conflict refers to the stress that arises when an individual faces two different roles of work and family, which are incompatible in some way, and when an individual has difficulty meeting the alternative demands of the roles. WFC and FWC are distinct but related forms of inter role conflict. From work-family and family-work perspectives, this type of conflict reflects the degree to which role responsibilities from the work and family domains are incompatible. Work-family conflict has a range of negative effects on nurses’ physical and mental health (14), as well as increasing work stress. A previous meta-analysis showed that work-family conflict has a significant positive effecting on nurses’ turnover intention (overall effect size of r=0.28, 95% CI [0.23−0.33]) (15). High levels of work-family conflict reduce job satisfaction and thus increase the turnover intention (16). A study of Chinese emergency nurses showed that marital status, years of work, and frequency of night shifts were correlates of work-family conflict (17), therefore, in the current study, we added the above variables as demographic factors influencing the willingness of nurses of different generations to leave their jobs.

Social support is an important factor in coping with stress in the work environment and is an important external resource that can be used from sources including family, friends, colleagues, etc. Perceived social support refers to the perception of support resources when needed (18). A study suggested that social support significantly reduces the stress of role conflict and moderates the relationship between work-family conflict and job performance (19). In the study of Saudi Arabian medical workers, social support was found to mediate the relationship between stress and turnover intentions, and social support was effective in mitigating the increase in turnover intentions associated with stress (20). In addition, perceived social support can also contribute to the reduction of job burnout (21). In a study during the epidemic, it was noted that perceived social support and the reduction of mental health problems helped to reduce the increased turnover of medical workers as a result of workplace violence (22).

Resilience is characterized by “when beset by problems and adversity, sustaining and bouncing back and even beyond to attain success” (23). The study of 350 nurses in Jilin province showed that resilience can indirectly influence turnover intention through job satisfaction and social support (24). Moreover, research on structural equation modeling for newly graduated nurses found that it is important to strengthen the resilience of new graduate nurses to reduce turnover intentions (25). Therefore, this study suggested that resilience may be associated with the turnover intention among nurses of different generations.

In this study, firstly, we aim to understand the turnover intention among the measured nursing population; secondly, to examine the differences in turnover intention across various demographic variables; and thirdly, to explore the influencing factors of nurses’ turnover intention based on generational differences, mainly considering the effects of perceived social support and resilience on nurses’ turnover intentions across generations. Based on the research findings, under general interventional measures, we propose corresponding preventive and interventional strategies tailored to the influencing factors specific to nurses of different generations.

## 2 Methods

### 2.1 Participants and procedure

We contacted the hospital leader or the director of nursing for consent before the questionnaire. From 4 September to 25 October 2023, a cross-sectional survey was performed among nurses at 16 tertiary hospitals in Jiangsu Province, China. Upon obtaining consent, we contacted the nursing departments of the target hospitals and randomly selected several departments to distribute questionnaire for the survey. The minimum sample size required for the study was calculated using the following formula: N= $\mu_{\alpha /2}^{2}*P*\left(1-P\right)/\delta^{2}$ , μ_α/2_ was the statistic for two-sided test with a confidence interval of 95%. P was the rate of nurses’ high turnover intention and δ was the permissible error. In our study, μ_α/2_ = 1.96. P=0.694, δ=0.03, considering the rate of invalid questionnaire, therefore the required size was 906. The survey was conducted by scanning the WeChat code and completed online through the “WenJuanXing” network platform. A total of 2,299 questionnaires were distributed and completed online, with 2,258 questionnaires returned. After applying the check-attention items (The items are designed without inherent significance, and participants are required to select the specified options) and exclusion standards, a total of 2,112 valid questionnaires were obtained, resulting in the efficiency of the questionnaire was 91.87%.

The inclusion criteria for participants included: 1) those who are certified as nurses and work in hospitals; 2) having no cognitive impairment and being able to understand the questions on the questionnaire; 3) voluntarily participating in our survey and following the principle of informed consent. The exclusion criteria were: 1) those who refused to participate, 2) the response time for completing the questionnaire exceeded standard deviation, and 3) the final questionnaires with missing or incorrect items.

### 2.2 Measurement tools

The questionnaire was divided into two parts, the first being the demographic variables. Demographic variables include gender, work years, marital status, the number of children to raise and elderly people to raise, educational level, whether they had an occupational disease, and number of night shifts. Second, the survey involved four scales as assessment tools:

#### 2.2.1 Work-family conflict scale

The scale was designed by Netemeyer et al. (26) and the Cronbach’s work-to-family conflict (WFC) and family-to-work conflict (FCW) were 0.88 and 0.82, respectively. The questionnaire consists of two parts: Questions 1–5 represent WFC, while questions 6–10 represent FWC conflict. The scale uses a Likert 4-point rating scale, with 1 indicating “strongly disagree” and 4 indicating “strongly agree”. The scale’s scoring range is from 10 to 40. A higher score indicates a higher level of conflict, while a lower score indicates a lower level of conflict. This scale has been utilized within the Chinese nursing population (27). In our study, the Cronbach’s α of WFCS is 0.910, subscales of WFC and FWC are 0.926 and 0.892, repectively.

#### 2.2.2 Multidimensional scale of perceived social support

The scale was developed by Zimet et al. (28) and Cronbach’s coefficient was 0.90. It consists of three dimensions: family support, friend support, and other support, with a total of 12 items. It uses a 7-point rating scale, and the minimum score obtainable is 12, while the maximum score is 84. A higher score indicates a higher level of perceived social support. The research of Liu et al. indicated that the scale demonstrates favorable measurement efficacy within the population of registered nurses in China (21). In the study, the Cronbach’s α of MSPSS is 0.952.

#### 2.2.3 Resilience questionnaire

The resilience scale was extracted from a psychological capital questionnaire developed by Luthans et al. (29) and the reliability of resilience subscale was 0.72 in Luthan’s research. Luo et al. (30) modified the scale based on the characteristics of nursing professionals in China. The questionnaire consists of 5 items and the range is from 5 to 30. The scale has exhibited good internal consistency in nurses (31). It uses a Likert 6-point scoring system, with scores ranging from 1 to 6, indicating “strongly disagree”, “disagree”, “somewhat disagree”, “somewhat agree”, “agree” and “strongly agree”, respectively. The Cronbach’s resilience is 0.909.

#### 2.2.4 Turnover intention questionnaire

The questionnaire was developed by Michael and Spector (32) and consists of 6 items and is scored on a 1–4 scale. The scale consists of 6 items, divided into 3 dimensions based on the relevance of the items. Items 1 and 6 are the possibility of quitting the current job (turnover intention I), the motivation of finding other jobs (turnover intention II) for items 2 and 3, and the possibility of getting other external jobs (turnover intention III) for items 4 and 5. The scale’s scoring range is from 6 to 24. A higher total score indicates a stronger intention to leave. Based on the scores and referencing the study by Liu et al. (4), a total mean score of less than or equal to 2 indicates low turnover intention, while a score greater than 2 indicates high turnover intention. In our study, Cronbach’s α of TIQ is 0.835.

### 2.3 Data analysis

Descriptive statistics analysis was initially performed to understand the impacts of various factors on turnover intention. Group differences were assessed using t-tests or analysis of variance (ANOVA). Pearson correlation analysis was utilized to evaluate significant variable relationships. Multiple linear regression analysis was also employed to identify the factors influencing nurses’ turnover intention across different generations. A significance level of P<0.05 was used to indicate statistical significance. All analyses were conducted using SPSS v26.0.

## 3 Results

Among the 2,112 participants, the majority were women (N = 2016, 95.45%). Marital status was primarily married (N = 1611, 76.28%). Most of them had one child or more (N = 1521, 72.0%) and had to raise the elderly (N = 1774, 84.00%). The work years were mainly below 10 years (N = 1009, 47.77%) or 11~20 years (N = 851, 40.29%). Most of them had graduated from university with a bachelor’s degree (N = 1898, 89. 87%). The types of employment were mainly contract systems (N = 1771, 83.85%). About half of the nurses (N = 995, 47.11%) had occupational disease. Mostly, night shifts for them every week were 0~1 times (N = 872, 41.29%) or 2~3 times (N = 860, 40. 72%) (**Table 1**).

**Table 1:** Comparison of scores of nurses with different demographic characteristics.

	Variables	N(%)	Turnover Intention			Work-family conflict			Social support			Resilience		
			*M±SD*	*T/F*	*P*	*M±SD*	*T/F*	*P*	*M±SD*	*T/F*	*P*	*M±SD*	*T/F*	*P*
Gender	Male	96(4.55)	13.14±4.64	-1.213	0.225	22.84±7.59	0.526	0.600	61.15±15.59	-2.586	0.010	23.05±4.99	0.384	0.702
	Female	2016(95.45)	13.64±3.99			22.43±6.42			64.67±12.09			22.85±4.37		
Work years	≤10	1009(47.77)	13.98±4.01	23.612	<0.001	22.52±6.57	7.690	<0.001	64.32±13.13	2.699	0.044	22.46±4.58	10578	<0.001
	11–20	851(40.29)	13.76±4.02			22.88±6.41			64.31±13.43			22.98±4.25		
	21–30	186(8.81)	12.08±3.58			20.83±5.95			64.84±11.10			23.70±3.92		
	≥31	66(3.13)	10.80±3.35			20.30±6.83			68.92±11.40			25.00±3.77		
Marital status	Unmarried	450(21.31)	14.04±4.01	3.175	0.042	22.29±6.37	0.601	0.548	63.47±12.81	4.809	0.008	22.20±4.52	6.723	0.001
	Married	1611(76.28)	13.51±4.01			22.47±6.51			64.93±13.08			23.03±4.35		
	Divorced/ Widowed	51(2.41)	13.35±4.16			23.31±6.39			60.39±13.53			23.33±4.45		
Number of children	0	591(27.98)	14.14±3.91	7.819	<0.001	22.24±6.26	8.877	<0.001	63.28±12.80	4.692	0.009	22.13±4.46	11.747	<0.001
	1	1014(48.01)	13.32±4.09			22.05±6.64			65.33±12.87			23.19±4.45		
	≥2	507(24.01)	13.64±3.96			23.49±6.30			64.29±13.61			23.06±4.13		
Raise the elderly	Yes	1774(84.00)	13.57±4.03	-1.312	0.190	22.59±6.44	2.298	0.022	64.45±13.08	-0.487	0.626	22.88±4.39	0.492	0.623
	No	338(16.00)	13.88±3.97			21.71±6.23			64.82±12.96			22.75±4.43		
Educational level	Associate degree & below	186(8.81)	12.69±4.21	6.173	0.002	21.44±7.38	2.474	0.085	62.65±13.96	2.697	0.068	23.28±4.51	1.153	0.316
	Bachelor’s degree	1898(89.87)	13.70±3.99			22.55±6.38			64.73±12.94			22.83±4.38		
	Master’s degree & higher	28(1.33)	14.61±4.07			22.46±6.003			61.96±13.99			22.29±4.40		
Type of employment	Career establishment	317(15.01)	12.19±3.84	24.271	<0.001	21.60±6.20	3.352	0.035	64.59±12.84	0.544	0.580	23.40±4.54	2.815	0.060
	Contract system	1771(83.85)	13.87±4.00			22.59±6.52			64.53±13.06			22.76±4.37		
	Labor dispatch system	24(1.14)	14.17±4.00			23.29±6.35			61.75±15.50			23.04±3.77		
Occupational disease	Yes	995(47.11)	14.67±3.89	11.625	<0.001	24.15±6.03	11.789	<0.001	62.13±13.43	-7.971	<0.001	22.21±4.46	-6.498	<0.001
	No	1117(52.89)	12.69±3.90			20.93±6.49			66.62±12.34			23.44±4.26		
Number of night shifts	0-1	872(41.29)	12.85±3.85	34.667	<0.001	21.61±6.25	21.192	<0.001	65.48±12.36	11.526	<0.001	23.25±4.26	8.301	<0.001
	2-3	860(40.72)	13.89±4.02			22.54±6.60			64.76±13.39			22.77±4.33		
	>3	380(17.99)	14.78±4.06			24.17±6.48			61.69±13.49			22.18±4.40		
Income/month	Below 3000	111(5.26)	14.15±4.58	7.271	<0.001	22.76±6.81	0.862	0.486	63.79±13.11	3.884	0.004	22.50±4.51	1.146	0.333
	3000-5000	408(19.32)	14.33±4.03			22.82±6.77			62.75±13.93			22.53±4.66		
	5001-7000	626(29.64)	13.79±3.91			22.24±6.58			65.19±12.28			22.96±4.38		
	7001-9000	538(25.47)	13.14±4.02			22.59±6.49			64.03±13.49			23.07±4.26		
	Above 9001	429(20.31)	13.16±3.86			22.14±6.48			65.96±12.54			22.87±4.29		

There were 243 (11.5%) nurses who were divided as “Generation X” between 43 to 58 old-age, 1544 (73.1%) nurses as “Generation Y” between 27 and 43 years old, and 325 (15.4%) nurses belonged to “Generation Z” aged 18-26 (**Table 2**).

**Table 2:** Important variables scores of nurses in different generations.

	N (%)	TIQ	LSD	WFC	LSD	SS	LSD	Resilience	LSD
Generation X①	243 (11.5)	1.93±0.59	①<②, p<0.001	20.94±6.20	①<②, p<0.001	65.49±11.99	①>②, p=0.232	23.99±3.83	①>②, p<0.001
Generation Y②	1544 (73.1)	2.32±0.67	①<③, p<0.001	22.73±6.48	①<③, p=0.018	64.41±13.34	①>③, p=0.264	22.76±4.41	①>③, p<0.001
Generation Z③	325 (15.4)	2.29±0.67	②>③, p=0.453	22.23±6.52	②>③, p=0.203	64.25±12.43	②>③, p=0.843	22.52±4.61	②>③, p=0.376
		F=36.292, P<0.001, ②>③>①		F=8.325, P<0.001, (2)>(3)>(1)		F=0.791, P=0.454, ①>②>③		F=9.524, P<0.001, ①>②>③	

TIQ, turnover intention questionnaire; WFC, work-family conflict; SS, social support.

Moreover, we found that the main difference existed between “Generation X” and the other two eras through post-facto comparison. The turnover intention in “Generation X” was lower than in “Generation Y” and “Generation Z” (P < 0.001). There were no significant differences in the turnover intention between “Generation Y” and “Generation Z” (**Table 2**).

There was a significant discrepancy between work years (P<0.001), marital status(P=0.042), amount of child (P <0.001), educational level (P = 0.002), type of employment (P<0.001), the number of night shifts among nurses (P<0.001) and income (P<0.001) for turnover intention. Nurses without children have more turnover intention than nurses with children (P<0.001). Nurses with occupational diseases are more likely to change jobs than nurses without occupational diseases (P<0.001) (**Table 1**). **Table 3** showed that different aspects of work-family conflict were positively related to turnover intention (P<0.01); family support, friend support, other support, and resilience were negatively related to the intention to turnover (P<0.01).

**Table 3:** Correlation analysis between important variables.

	1	2	3	4	5	6	7
1 Work-family conflict	1.000						
2 Family-work conflict	0.501^**^	1.000					
3 Family support	-0.297^**^	-0.323^**^	1.000				
4 Friend support	-0.313^**^	-0.320^**^	0.733^**^	1.000			
5 Other support	-0.376^**^	-0.312^**^	0.711^**^	0.793^**^	1.000		
6 Resilience	-0.334^**^	-0.210^**^	0.396^**^	0.439^**^	0.457^**^	1.000	
7 Turnover intention	0.453^**^	0.321^**^	-0.323^**^	-0.312^**^	-0.374^**^	-0.336^**^	1.000
Mean	12.960	9.489	21.921	21.866	20.720	22.862	13.622
SD	3.926	3.548	4.865	4.481	4.983	4.397	4.020

^**^Correlation is significant at the .01 level (1-tailed).

**Table 4** depicts the multiple linear regression of impact factors associated with turnover intention based on intergenerational differences. Multiple linear regression analysis was carried out with the aggregate turnover intention of nurses of different generations divided into dependent variables, factors with statistical significance in the univariate analysis as independent variables, the two aspects of work-family conflict, and the three aspects of perceptive social support and resilience as explanatory variables. Work-family conflict (β= 0.196, P = 0.009) positively associated with switching nurses from “Generation X”. Night shifts (β= 0.047, P = 0.033), work-family conflict (β = 0.283, P<0.001), and family-work conflict (β = 0.099, P<0.001) were positively associated with “Generation Y” nurse intention to leave. Occupational disease (β = -0.108, P<0.001), other support (β = -0.169, P<0.001), the number of children (β = -0.054, P = 0.041) and resilience (β = -0.103, P<0.001) were negatively associated with the intention of “Generation Y” nurses to leave. Work-family conflict (β = 0.311, P<0.001) was positively associated with the intention of “Generation Z” nurses to leave. Family support (β = -0.209, P = 0.010) and resilience (β = -0.188, P = 0.001) were negatively associated with the intention of “Generation Z” nurses to leave.

**Table 4:** Multiple linear regression analysis of influencing factors of demographic characteristics on turnover intention of nurses of different generations.

Category	Variable	β	t	P	95%CI (LL)	95%CI (UL)
Generation X	Constant	–	1.497	0.136	-0.578	4.237
	WFC	0.196	2.632	0.009	0.045	0.310
Generation Y	Constant	–	-0.743	0.458	-1.049	0.473
	Number of children	-0.054	-2.044	0.041	-0.153	-0.003
	Occupational Diseases	-0.108	-4.839	<0.001	-0.302	-0.058
	Night shifts	0.047	2.137	0.033	0.005	0.126
	WFC	0.283	10.641	<0.001	0.228	0.332
	FWC	0.099	3.918	<0.001	0.049	0.149
	Other support	-0.169	-4.452	<0.001	-0.235	-0.091
	Resilience	-0.103	-4.093	<0.001	-0.151	-0.053
Generation Z	Constant	–	0.599	0.550	-1.539	2.885
	WFC	0.311	4.886	<0.001	0.115	0.449
	Family support	-0.209	-2.589	0.010	-0.310	-0.076
	Resilience	-0.188	-3.418	0.001	-0.203	-0.063

WFC, work-family conflict; FWC, family-work conflict.

a *R* ^2^=0.171, adjusted *R* ^2^=0.112, *F*=22.908, *P*<0.001;

b *R* ^2^=0.314, adjusted *R* ^2^=0.307, *F*=43.676, *P*<0.001;

c *R* ^2^=0.353, adjusted *R* ^2^=0.320, *F*=10.510, *P*<0.001.

## 4 Discussion

Improving nurse retention rates is a crucial objective globally. This study explores the factors influencing job-quitting intentions among nurses of different generations. It offers nursing managers targeted strategies to mitigate nurse turnover to improve patient care quality. Our findings reveal that the intention to leave among nurses is high, as 60.2% of participants have a high turnover intention. This is consistent with the research in Hunan province by Yang Liu et al., where 69.4% of nurses reported a high intention to leave (4). The average turnover rate of nurses in secondary hospitals is higher than that in tertiary hospitals, because nurses in tertiary hospitals enjoy a better working environment and enhanced career development opportunities (33).

The turnover intention in “Generation X” was lower than in “Generation Y” and “Generation Z”. In China, “Generation X” people were born in a period characterized by relatively limited access to material resources, experiencing slow social mobility and a moderate pace of cultural transformation, which fostered their resilient personalities and relatively conservative attitudes toward life. Significantly different from “Generation X” nurses, “Generation Y” was born in the reform era and grew up with the trend of reform and opening up. They are more inclined to open ideas and pay more attention to career aspirations. “Generation Z” grew up in the 21st century, experiencing the same turnover intention as “Generation Y” nurses in the same era characteristics.

This study also found that common factors affect nurses’ turnover intention in different generations. Work-family conflict is a factor influencing the turnover intention of nurses from the “Generation X”, “Generation Y” and “Generation Z” groups. Compared to other professions, the nursing profession is constantly exposed to high-pressure situations, with irregular daily schedules (34). In addition, the shortage of nursing human resources leads to greater workloads for nurses, requiring them to invest more time and energy into their work. At the same time, the medical institutions involved in this survey are all tertiary medical institutions and the educational levels of nursing staff are mostly bachelor’s degree or above. Research has shown that individuals with higher education levels have higher levels of work engagement and intrinsic motivation (35), leading to higher levels of involvement in work after hours. The more one is engaged in work, the less they tend to invest in their family. When nurses find it difficult to balance work and personal life, uncertainty arises, leading to emotional exhaustion and stimulating intentions to leave the job (36). When personnel in the medical profession face work-family conflict, reviewing conflicts between work and family to identify mutually beneficial strategies for labor and management becomes necessary (37). Therefore, nurse managers can maintain sensitivity and empathy towards employees’ work-family issues by providing appropriate guidance on the organization’s work-family policies (38) and offering organizational support to nurses, including adequate staffing and flexible work schedules, to better assist nurses in achieving a balance between their work and family roles, and then reduce nurses’ turnover intention (39).

In addition to the common influencing factors mentioned above, this study found that there are specific factors that affect the turnover intention of the nurse groups of “Generation X”, “Generation Y” and “Generation Z”. Resilience, the number of children, occupational diseases, night shift frequency, family-work conflict, and other supports are specific influencing factors that affect the turnover intention of “Generation Y” nurses. Previous studies have shown a significant negative correlation between resilience and turnover intention (24, 25). “Generation Y” is the backbone of nursing teams in various hospitals, with most of them having worked for more than 10 years. When faced with adversity at work, they will find ways to overcome difficulties, do their part as well as possible, and increase their work engagement to reduce turnover intention (40). At the same time, these nurses bear the main family responsibilities, also being in a period of professional advancement and facing high job pressures. Nurses are more susceptible to musculoskeletal disorders due to the nature of their work, as supported by research by Fronteira et al. (41). Specifically, nurses often need to maintain a bent posture for long periods while performing nursing tasks such as drawing blood, dispensing medications, and observing drainage. The annual incidence rate of low back pain among nurses is on average 70% (42–44). Therefore, for the sake of their own health and family companionship, Gen Y nurses may have a higher intention to leave the workforce.

Previous studies have shown that frequent night shifts not only affect the physical health of nurses, leading to insufficient sleep and posing obstacles to shift work (45) but also affect the family and social lives of nursing staff, disrupting their normal family life, including the inability to fully care for children (46, 47). However, female nurses still dominate the clinical nursing field in China. Influenced by traditional family culture, women usually take on more responsibilities in raising children and caring for the elderly (48). Currently, “Generation Y” nursing staff are at a critical stage of child-rearing, making them unable to fully dedicate themselves to work as they strive to balance their roles and responsibilities at work and home. These issues give rise to work-family and family-work conflicts and may sometimes prompt nurses to consider resigning. When discussing the factors influencing nurses’ turnover intention, one crucial aspect that cannot be overlooked is their subjective feelings. Nurses are more willing to remain in their positions when they feel cared for and helped by colleagues, as well as understood and respected by hospital managers (49). Conversely, if these needs are not met, their intention to leave is higher. Therefore, it is evident that healthcare institutions should endeavor to foster a unified and supportive work atmosphere, enabling nurses to experience care and a profound sense of belonging in their work environment.

Resilience and family support are specific influencing factors of the turnover intention of “Generation Z” nurses (50). “Generation Z” has high expectations for a healthy work-life balance, since they value workplace flexibility and autonomy. Due to their age, there are instances where young nurses feel that they are not respected or are treated like children (51). Therefore, when the actual work does not meet their expectations, thoughts of leaving the job are more likely to arise. Therefore, nursing managers should gain a thorough understanding of the career development expectations of nurses with different educational backgrounds. This will enable them to provide more targeted development opportunities, such as positions for specialized nurses, clinical instructors, and research nurses. By doing so, they can better meet the career aspirations of nurses and promote the stable development of the nursing team (52). At the same time, the “Generation Z” nurse group, being younger than 35 years old, typically has less than 10 years of work experience. As a result, they may lack the experience of balancing roles between work and family. Some nurses may find it challenging to cope with the pressure stemming from changes in responsibilities and additional roles they take on after marriage or other life events (53). Understanding and support from family members effectively alleviate their concerns about the future, strengthening their resolve to stay in the profession and reducing their willingness to resign. On the contrary, nurses with inadequate family support may experience more negative emotions when facing job stress, which may in turn affect their intention to leave. Therefore, healthcare institutions and management departments should pay attention to the family situation of young nurses, improve the working environment, enhance benefits, and increase nurses’ family satisfaction levels to reduce turnover intention.

It can be seen that “Generation X” nurses are more focused on family than job development opportunities. When work becomes a problem for their families, they may want to leave. While “Generation Y” nurses are in a critical period of career development, for them, work-related factors have more influence on their turnover intention. “Generation Z” nurses are new to their jobs and are more focused on themselves. Family support can effectively increase their willingness to stay.

This paper exhibits several limitations that warrant attention. Due to the inherent nature of correlation and causation coexisting, the inferential power of causality in our cross-sectional survey is notably weak. We surveyed 16 hospitals in Jiangsu Province, covering most areas of the province, but our sample was limited to tertiary hospitals. The conclusions cannot be extrapolated to community and township hospitals. This study was also subject to monomethod and self-report bias as all measures were nurse-reported and collected via an online survey. In the future, the sample sampling scope should be further expanded to lower-level hospitals in county and township, and the sampling should be expanded to other provinces in the country. A follow-up survey can also be adopted to establish a research cohort.

## 5 Implications for practice

In conclusion, turnover intention status and influencing factors among nurses from different generations vary due to the influences of growth environments, life experiences, and values. These differences requires nursing managers to adopt more detailed and comprehensive strategies in their management practices. These strategies should address and resolve various potential risks related to nursing turnover. Work-family conflict is a common factor influencing the turnover intention among nurses across different generations. When nurses encounter inevitable conflicts between work and family, the hospital administration should proactively coordinate to minimize the occurrence of work-family conflicts. In cases where such conflicts have already arisen, the supervising leaders of the respective departments should provide psychological comfort and necessary material support. and counseling to alleviate the internal pressure on the nurses. Luthans has stated that resilience is an individual's internal resource, a trait-like, and can be cultivated. Therefore, hospitals can appropriately conduct psychological lectures to disseminate relevant knowledge, enabling nurses to learn how to alleviate the stress caused by work-family conflicts. Furthermore, hospitals should establish systems to safeguard the physical health of their staff, aimed at reducing the negative impact of occupational diseases on employees. The families of Gen Z nurses should offer more understanding and support for their work to alleviate the psychological pressure arising from the conflict between work and family caused by their work.

## 6 Conclusions

This study found that work-family conflict was a common factor affecting nurses of different generations. When the conflict between the two has an impact on the nurse’s family, the manager should intervene in time to reconcile the conflict. In addition, there are different factors affecting the turnover intention of nurses in different generations. This requires nurse managers to consider the differences between different generations of nurses when effectively formulating measures to reduce turnover intention.

## Data Availability

The original contributions presented in the study are included in the article/supplementary material. Further inquiries can be directed to the corresponding author/s.
